# D-Dimer Levels Predict Myocardial Injury in ST-Segment Elevation Myocardial Infarction: A Cardiac Magnetic Resonance Imaging Study

**DOI:** 10.1371/journal.pone.0160955

**Published:** 2016-08-11

**Authors:** Soonuk Choi, Woo Jin Jang, Young Bin Song, Joao A. C. Lima, Eliseo Guallar, Yeon Hyeon Choe, Jin Kyung Hwang, Eun Kyoung Kim, Jeong Hoon Yang, Joo-Yong Hahn, Seung-Hyuk Choi, Sang-Chol Lee, Sang Hoon Lee, Hyeon-Cheol Gwon

**Affiliations:** 1 Division of Cardiology, Department of Medicine, Heart Vascular Stroke Institute, Samsung Medical Center, Sungkyunkwan University School of Medicine, Seoul, Republic of Korea; 2 Division of Cardiology, Department of Internal Medicine, Samsung Changwon Hospital, Sungkyunkwan University School of Medicine, Changwon, Republic of Korea; 3 Department of Epidemiology, Johns Hopkins Bloomberg School of Public Health, Baltimore, Maryland, United States of America; 4 Department of Medicine, Johns Hopkins University School of Medicine, Baltimore, Maryland, United States of America; 5 Department of Radiology, Cardiovascular Imaging Center, Heart Vascular Stroke Institute, Samsung Medical Center, Sungkyunkwan University School of Medicine, Seoul, Republic of Korea; GERMANY

## Abstract

**Objectives:**

Elevated D-dimer levels on admission predict prognosis in patients undergoing primary percutaneous coronary intervention (PCI) for ST-segment elevation myocardial infarction (STEMI), but the association of D-dimer levels with structural markers of myocardial injury in these patients is unknown.

**Methods:**

We performed cardiac magnetic resonance (CMR) imaging in 208 patients treated with primary PCI for STEMI. CMR was performed a median of 3 days after the index procedure. Of the 208 patients studied, 75 patients had D-dimer levels above the normal range on admission (>0.5 μg/mL; high D-dimer group) while 133 had normal levels (≤0.5 μg/mL; low D-dimer group). The primary outcome was myocardial infarct size assessed by CMR. Secondary outcomes included area at risk (AAR), microvascular obstruction (MVO) area, and myocardial salvage index (MSI).

**Results:**

In CMR analysis, myocardial infarct size was larger in the high D-dimer group than in the low D-dimer group (22.3% [16.2–30.5] versus 18.8% [10.7–26.7]; p = 0.02). Compared to the low D-dimer group, the high D-dimer group also had a larger AAR (38.1% [31.7–46.9] versus 35.8% [24.2–45.3]; p = 0.04) and a smaller MSI (37.7 [28.2–46.9] versus 47.1 [33.2–57.0]; p = 0.01). In multivariate analysis, high D-dimer levels were significantly associated with larger myocardial infarct (OR 2.59; 95% CI 1.37–4.87; p<0.01) and lower MSI (OR 2.62; 95% CI 1.44–4.78; p<0.01).

**Conclusions:**

In STEMI patients undergoing primary PCI, high D-dimer levels on admission were associated with a larger myocardial infarct size, a greater extent of AAR, and lower MSI, as assessed by CMR data. Elevated initial D-dimer level may be a marker of advanced myocardial injury in patients treated with primary PCI for STEMI.

## Introduction

D-dimer is a degradation product of cross-linked fibrin with established clinical utility for diagnosing pulmonary embolism and deep vein thrombosis [1] and could be also one of the useful biomarkers for acute myocardial infarction (MI) because ruptured plaque-induced coronary thrombus plays an important role in the pathophysiology of acute MI [2]. In addition, an elevated level of D-dimer is associated with subsequent cardiovascular events in apparently healthy men and patients with unstable angina [3–5]. Recently, elevated D-dimer levels on admission were found to predict both adverse cardiovascular events and major bleeding in patients with ST-segment elevation myocardial infarction (STEMI) undergoing primary percutaneous coronary intervention (PCI) [6]. Another study evaluating the prognostic value of D-dimer in STEMI patients showed that elevated D-dimer levels were associated with in-hospital and mid-term mortality [7]. Currently, no attempt has been made to evaluate the impact of D-dimer levels on myocardial injury and salvage in STEMI patients. Cardiac magnetic resonance (CMR) imaging can accurately quantify myocardial ischemic injury and salvaged myocardium, providing a better understanding of the impact of D-dimer in STEMI patients [8, 9]. Therefore, we evaluated the association between D-dimer levels on admission and markers of myocardial injury using CMR in STEMI patients undergoing primary PCI.

## Materials and Methods

### Study Population

**Fig 1:**
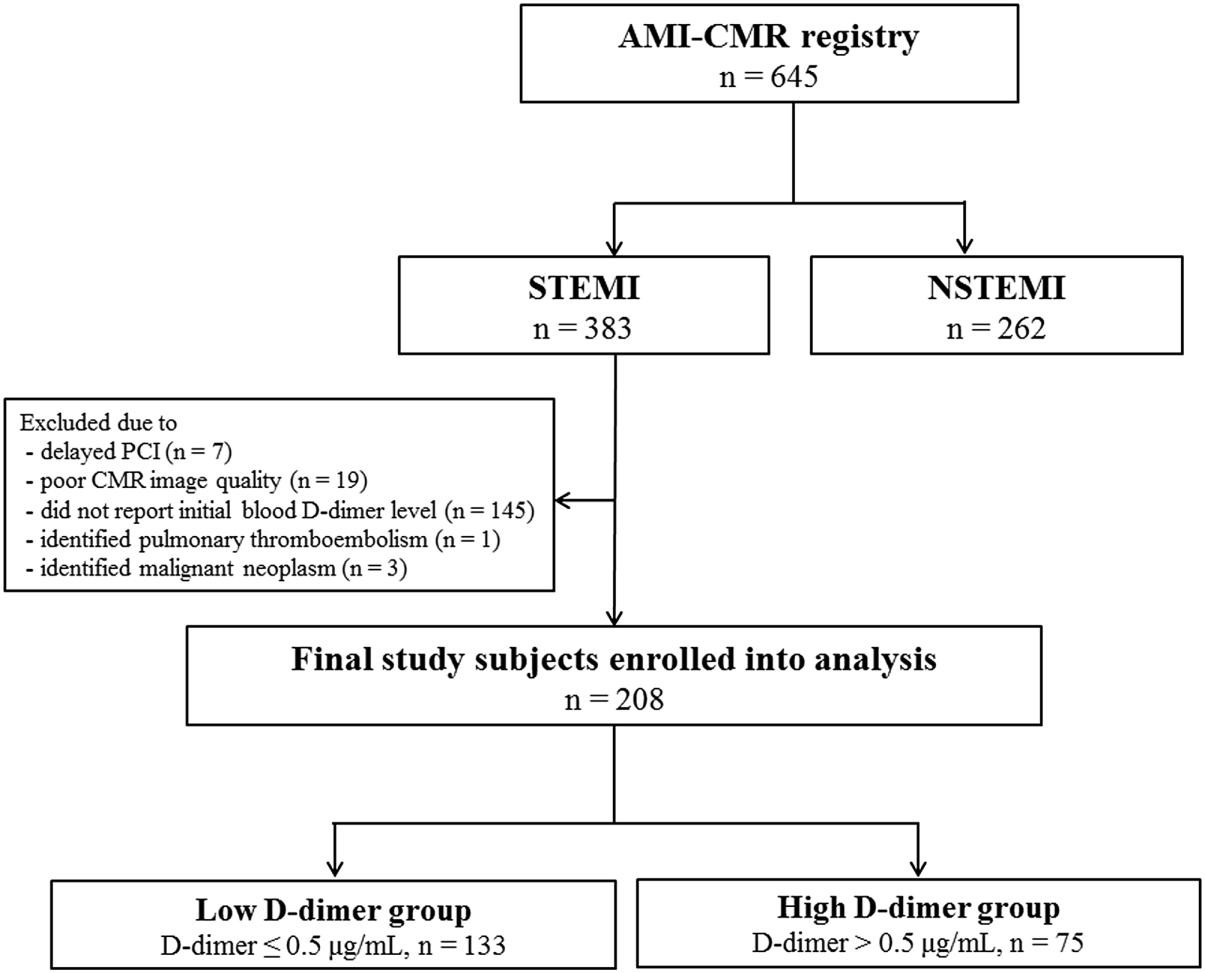
Scheme of Group Distribution. AMI-CMR = acute myocardial infarction–cine magnetic resonance imaging; STEMI = ST-segment elevation myocardial infarction; NSTEMI = non ST-segment elevation myocardial infarction; PCI = percutaneous coronary intervention; CMR = cardiac magnetic resonance imaging.

A detailed description of the study methods is presented in the Supplementary Appendix (S1 File). The study population consisted of patients on the Acute Myocardial Infarction–Cine Magnetic Resonance imaging (AMI-CMR) registry at Samsung Medical Center in Seoul, Korea, from December 2007 to July 2014 (n = 645). We only included patients with STEMI and excluded patients with the following: a history of coronary artery bypass grafting, thrombolysis for acute MI, or cardiopulmonary resuscitation; poor-quality CMR data (n = 19); or a history of pulmonary thromboembolism (n = 1) or malignancy (n = 3). The final sample size for the study was 208 patients (161 men, 47 women, Fig 1). The Institutional Review Board of Samsung Medical Center approved this study, and all subjects provided written informed consent to participate in this study.

### Laboratory Analysis of D-dimer

We obtained blood samples in 3.2% sodium citrate tubes from all patients upon admission before any therapy. D-dimer levels were measured using an immunoturbidimetric assay and fibrinogen equivalent units. The immunoturbidimetric assay is a second-generation latex agglutination assay that records the rate at which antibody-coated particles aggregate in response to the D-dimer antigen using an automated method on specialized analyzers [10]. The normal range of D-dimer at our institution was 0 to 0.50 μg/mL, the analytical measurement range was 0.27 to 4.00 μg/mL, and the clinically reportable range was 0.01 to 60.00 μg/mL. Of the 208 patients included in the study, 75 patients had D-dimer levels above the normal range on admission (>0.5 μg/mL; high D-dimer group) while 133 had normal levels (≤0.5 μg/mL; low D-dimer group).

### Data Collection, Definitions, and Study Outcomes

The baseline characteristics, angiographic and procedural findings, and CMR data were recorded prospectively by research coordinators as part of a dedicated registry [11]. Killip classification was determined upon arrival or before primary PCI. STEMI was defined as ST-segment elevation ≥ 1 mm in two or more contiguous leads or newly developed left bundle branch block on electrocardiogram.

Before primary PCI, we obtained blood samples for the analysis of N-terminal pro B-type natriuretic peptide (NT-proBNP), creatine kinase-myocardial band isoenzyme (CK-MB), D-dimer, and uric acid in all study patients. Serum CK-MB levels were serially evaluated every 8 hours after the index procedure until a peak value was confirmed. Left ventricular ejection fraction (LVEF) was measured by transthoracic echocardiography using Simpson’s methods before or immediately after primary PCI.

Larger myocardial infarct size was defined as ≥20% of the median infarct size measured by CMR [12], and lower myocardial salvage index (MSI) was defined as <44 of the median MSI in the present study [13]. Multi-vessel disease was defined as stenosis >50% noted in more than two coronary arteries. Angiographic no-reflow was defined as impeded blood flow to the ischemic tissue after relief of the occlusion, based on a previous description [14].

The primary outcome was myocardial infarct size. The secondary outcomes were extent of area at risk (AAR), MSI, microvascular obstruction (MVO) area, left ventricular end-diastolic volume (LVEDV), left ventricular end-systolic volume (LVESV) and left ventricular ejection fraction (LVEF).

### Cardiac Magnetic Resonance Imaging Analysis

**Fig 2:**
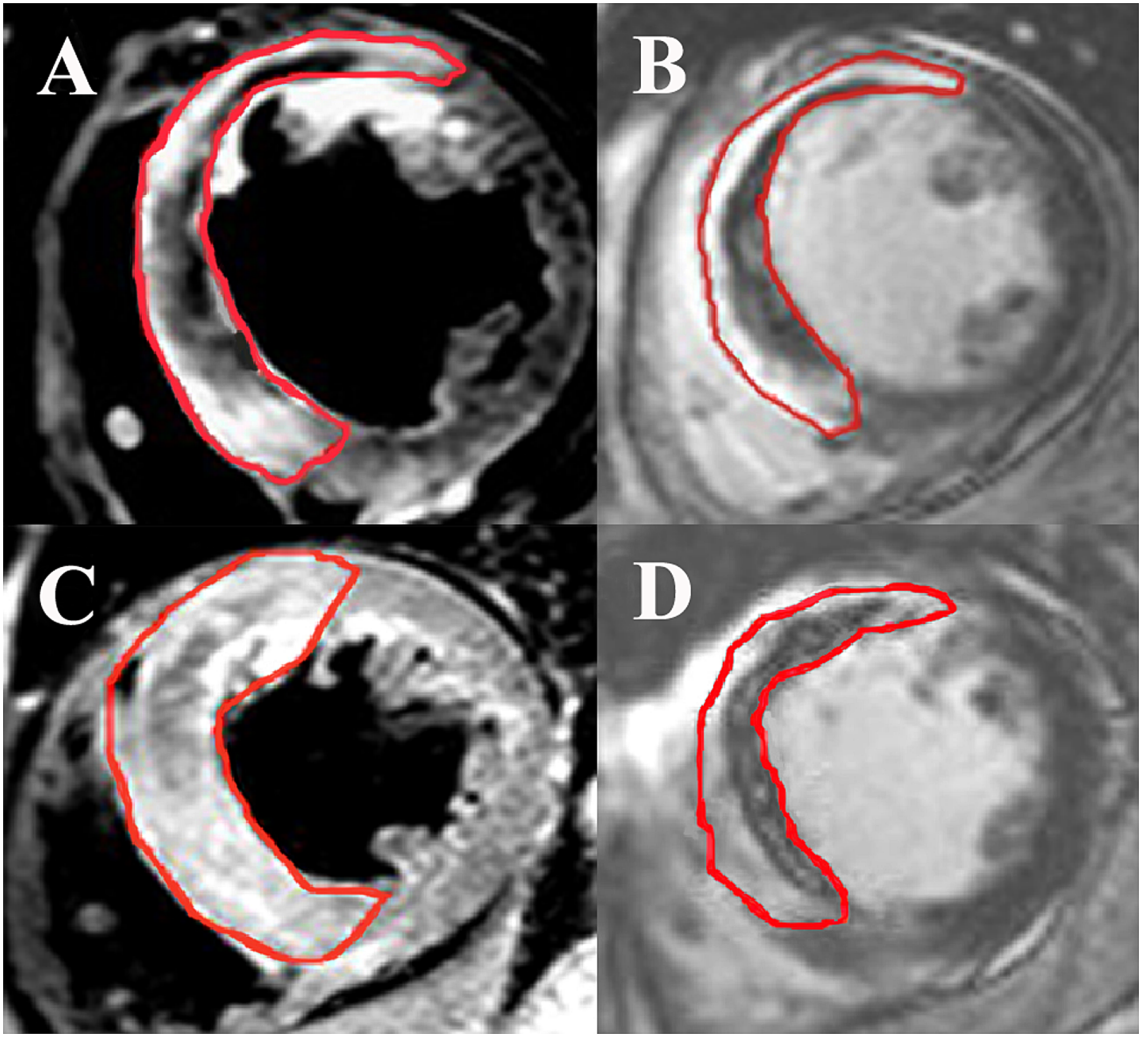
Example Images From a Study of Reperfused Anterior ST-segment Elevation Myocardial Infarction. Representative cardiac magnetic resonance images of a reperfused anterior ST-segment elevation myocardial infarction: (A) high D-dimer level with a short-axis slice of a T2-weighted image, (B) high D-dimer level with a late-gadolinium enhancement image, (C) low D-dimer level with a short-axis slice of a T2-weighted image, (D) low D-dimer level with a late-gadolinium enhancement image. In these cases, D-dimer levels, area at risk, and infarct size were 1.43μg/mL versus 0.26μg/mL, 72.2% versus 55.6% and 38.6% versus 17.9%, respectively, yielding a myocardial salvage index of 46.5 versus 67.8.

All measurements were made at Samsung Medical Center-CMR core laboratory using validated software (ARGUS, Siemens Medical System, Erlangen, Germany). Two experienced radiologists blinded to patient information performed these measurements. To avoid a partial volume effect, the size of myocardial enhancement was quantified using a threshold analysis with normalization to remote myocardium. Therefore, bright myocardium was measured as a region with a signal intensity more than 6 standard deviations above remote myocardium on T2-weighted images for AAR or delayed hyperenhancement images for infarct size [15]. After acquiring the short-axis images at end-diastole and end-systole, the endocardial borders were traced and Simpson’s algorithm was used to calculate the LVEDV, LVESV, and LVEF [16]. Infarct size and extent of MVO were assessed on delayed enhanced images. Infarct size was calculated from the summation of the area with delayed hyperenhancement within each segment of the short-axis images. This value was multiplied by slice thickness in order to cover the entire left ventricle. The endocardial and epicardial borders were planimetered to calculate the myocardial area. They were then summed to calculate the LV myocardial volume using the same method. Infarct size was expressed as percentage of affected LV myocardial volume. T2-weighted images were used to determine the presence of hemorrhagic infarction [17]. The AAR was quantified on T2-weighted images using a similar algorithm as above and was similarly expressed as percentage of LV myocardial volume affected. The MSI was calculated using the following formula: MSI = (AAR − infarct size) x 100/AAR [13]. Fig 2 shows a representative CMR image of reperfused anterior STEMI.

### Statistical Analysis

Continuous variables were summarized as mean ± SD or median and interquartile range (IQR) and were compared using independent *t*-tests or Wilcoxon rank sum tests. Categorical variables were compared with Pearson’s χ^2^ or Fisher’s exact tests. Multivariate logistic regression analysis was performed with a stepwise backward selection process to determine the independent predictors of large myocardial infarct and low MSI [11]. Clinical variables (age, gender, current smoker, diabetes mellitus, hypertension, and dyslipidemia) and possible determinants of infarct size (infarct location, number of diseased vessels, baseline thrombolysis in myocardial infarction [TIMI] flow grade, PCI history, manual thrombectomy during PCI, and use of glycoprotein IIb/IIIa inhibitor) were included in the regression models. The criteria for inclusion and exclusion of variables were set at 0.05 and 0.20, respectively. All tests were two-tailed, and p value <0.05 was considered statistically significant. Statistical analyses were performed with the SAS 9.2 (SAS Institute Inc., Cary, NC, USA) and were verified by Samsung Medical Center Statistics Support team (Samsung Biomedical Research Institute, Samsung Medical Center, Seoul, Korea).

## Results

### Baseline Clinical, Angiographic, and Procedural Characteristics

The baseline clinical, angiographic, and procedural characteristics of the patients stratified by D-dimer level are shown in Table 1. Compared to the low D-dimer group, patients in the high D-dimer group were older and more likely to have Killip class ≥2, multi-vessel disease, and higher levels of NT-proBNP. Patients in the high D-dimer group were less likely to be male or current smokers, and had a tendency to have a lower frequency of final myocardial blush grade 3 than patients in the low D-dimer group. In both groups, the most frequent culprit vessel was the left anterior descending artery and the baseline TIMI flow grade was close to 0. Other baseline characteristics were not significantly different between the two groups.

**Table 1:** Baseline Characteristics of Study Participants.

	Overall	Low D-dimer group ^a^	High D-dimer group ^a^	p value
	n = 208	n = 133	n = 75	
Age, years	60.0 (52.0–69.0)	56.0 (49.5–61.5)	70.0 (62.0–76.0)	<0.01
Male	161 (77.4)	111 (83.5)	50 (66.7)	0.01
Current smoker	91 (43.8)	72 (54.1)	9 (25.3)	<0.01
Diabetes mellitus	56 (26.9)	36 (27.1)	20 (26.7)	0.95
Hypertension	96 (46.2)	56 (42.1)	40 (53.3)	0.12
Dyslipidemia	36 (17.3)	22 (16.5)	14 (18.7)	0.70
Previous PCI	13 (6.3)	9 (6.8)	4 (5.3)	0.68
Killip class ≥2	25 (12.0)	9 (6.8)	16 (21.3)	<0.01
NT-proBNP ^b^	96.7 (19.3–430.8)	54.6 (14.7–316.3)	1429 (43.2–1203.0)	<0.01
Peak CK-MB	170.7 (61.4–288.9)	168.1 (67.8–275.4)	181.3 (46.8–300.0)	0.68
Culprit vessel				0.90
LAD	107 (51.4)	70 (52.6)	37 (49.3)	
LCx	21 (10.1)	14 (10.5)	7 (9.3)	
RCA	79 (38.0)	48 (36.1)	31 (41.3)	
Left main	1 (0.5)	1 (0.8)	0 (0)	
Multi-vessel disease	87 (41.8)	45 (33.8)	42 (56.0)	<0.01
TIMI 0, baseline	158 (76.0)	104 (78.2)	54 (72.0)	0.32
TIMI, final				0.74
TIMI 1, final	1 (0.5)	1 (0.8)	0 (0)	
TIMI 2, final	12 (5.8)	8 (6.0)	4 (5.3)	
TIMI 3, final	195 (93.8)	124 (93.2)	71 (94.7)	
No-reflow	13 (6.3)	9 (6.8)	4 (5.3)	0.68
Final MBG 3	192 (92.3)	126 (94.7)	66 (88.0)	0.08
Thrombus aspiration	137 (65.9)	87 (65.4)	50 (66.7)	0.86
Glycoprotein IIb/IIIa inhibitor	37 (17.8)	24 (18.0)	13 (17.3)	0.90

Values are expressed as median (interquartile range) or n (%).

^**a**^Low D-dimer group was defined as D-dimer ≤0.5 μg/mL and high D-dimer group was defined as D-dimer >0.5 μg/mL.

^**b**^NT-proBNP was available in 97.0% (n = 129) of patients in the high D-dimer group and in 98.7% (n = 74) of patients in the low D-dimer group.

PCI = percutaneous coronary intervention; NT-proBNP = N-terminal pro B-type natriuretic peptide; CK-MB = creatine kinase myocardial band isoenzyme; LAD = left anterior descending artery; LCx = left circumflex artery; RCA = right coronary artery; TIMI = thrombolysis in myocardial infarction flow grade; MBG = myocardial blush grade.

### Cardiac Magnetic Resonance Analysis

**Fig 3:**
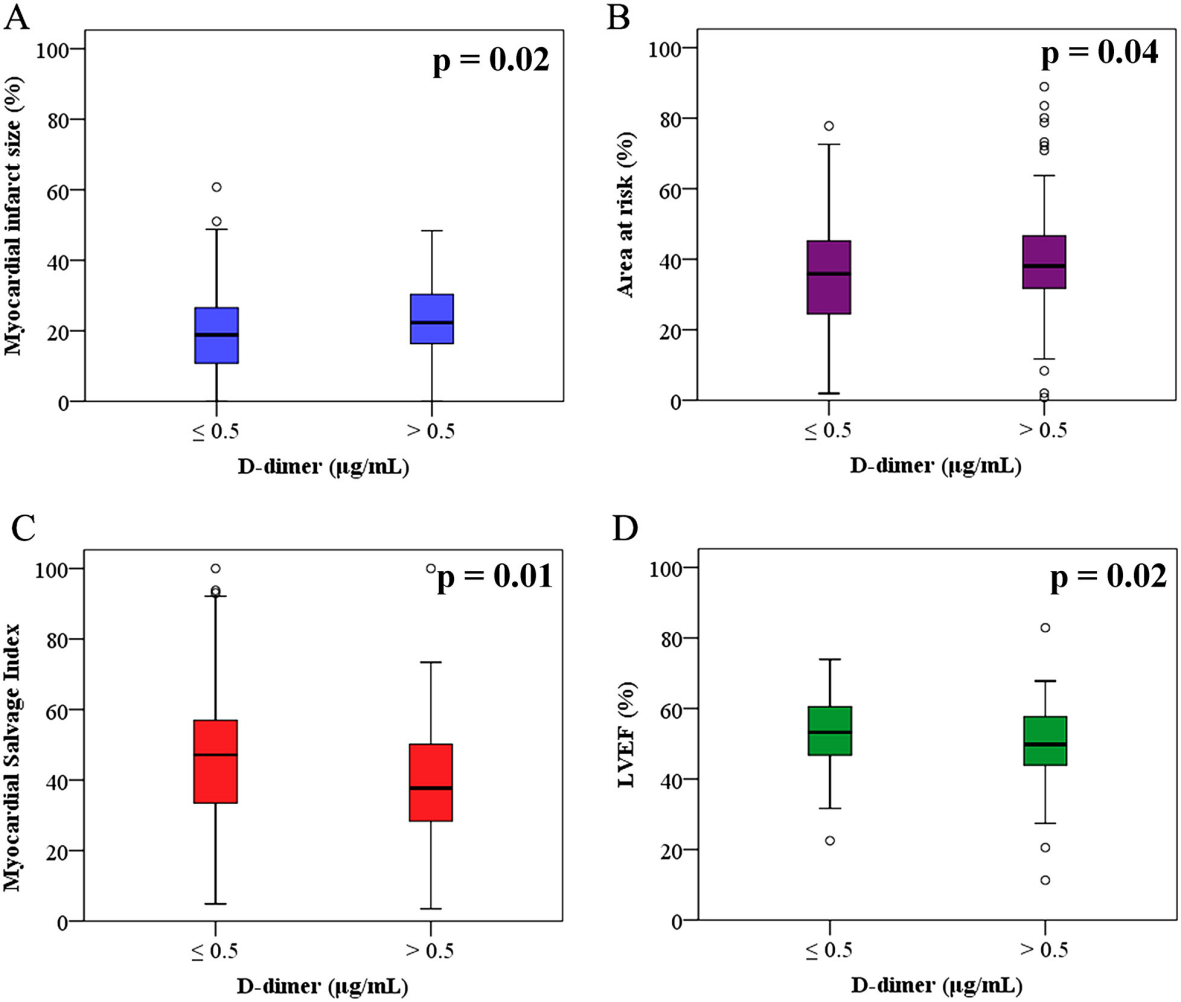
Cardiac Magnetic Resonance Findings According to D-dimer Level. Boxplots shows cardiac magnetic resonance data according to D-dimer level and (A) myocardial infarct size, (B) area at risk, (C) myocardial salvage index, and (D) LVEF.

**Fig 4:**
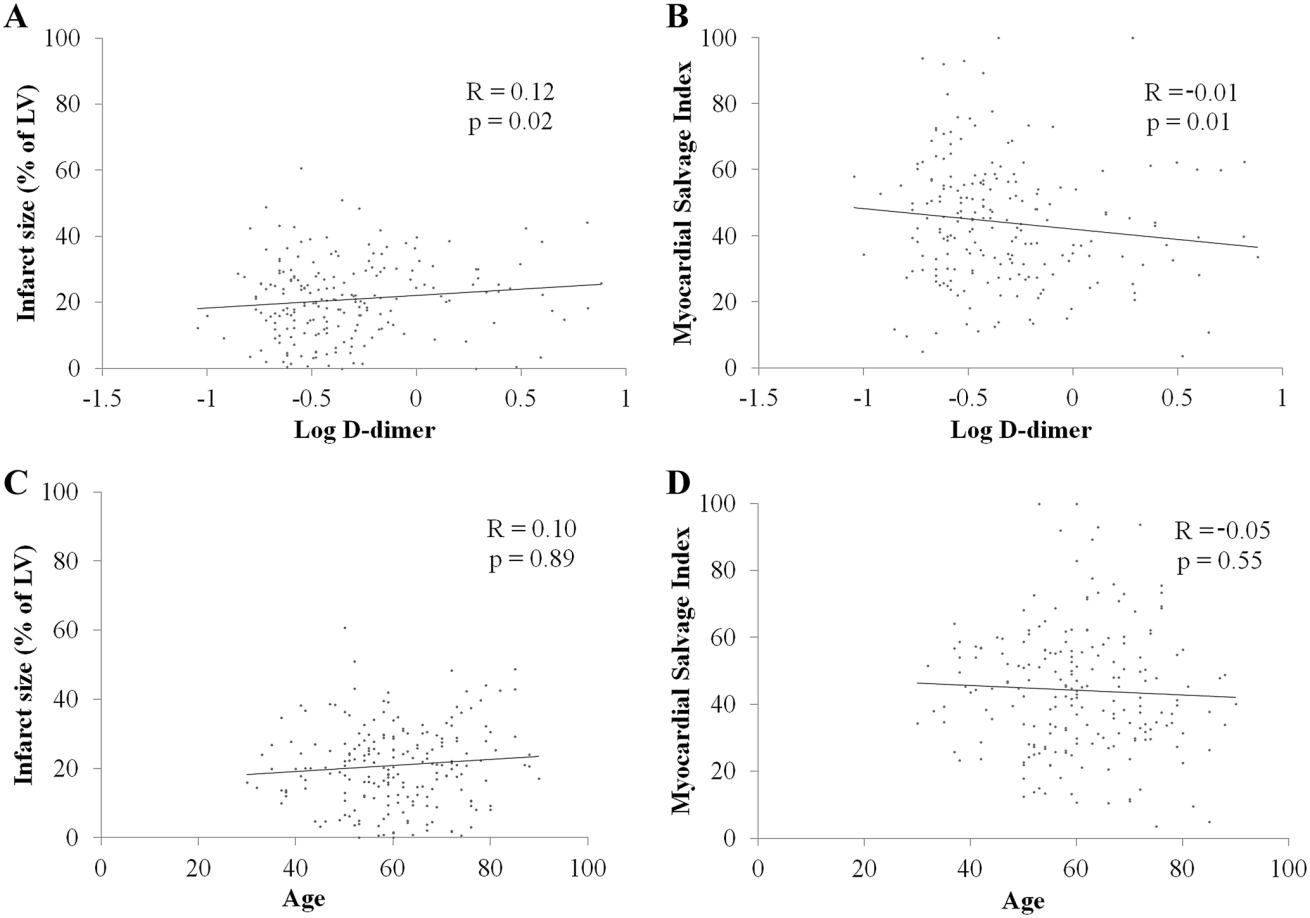
Linear regression analyses for Relationship Between Myocardial Injury, Level of D-dimer, and Age. (A) Relationship between D-dimer level and infarct size. (B) Relationship between D-dimer level and myocardial salvage index. (C) Relationship between age and infarct size. (D) Relationship between age and myocardial salvage index.

The results of CMR are presented in Table 2. CMR was performed a median of three days after the index procedure (IQR, three to four days) and there was no difference in the time interval from the procedure to CMR between the two groups (p = 0.23). Myocardial infarct size of the left ventricle (22.3% [16.2 to 30.5] versus 18.8% [10.7 to 26.7]; p = 0.02) was significantly larger and the AAR of the left ventricle (38.1% [31.7 to 46.9] versus 35.8% [24.2 to 45.3]; p = 0.04) was larger in the high D-dimer group than in the low D-dimer group. MSI (37.7 [28.2 to 46.9] versus 47.1 [33.2 to 57.0]; p = 0.01) and LVEF (49.8% [43.9 to 57.8] versus 53.3% [46.7 to 60.6]; p = 0.02) were lower in the high D-dimer group than in the low D-dimer group (Fig 3). However, the MVO area of the left ventricle, LVEDV, and LVESV were not statistically different between the two groups. In addition, the level of D-dimer was shown to be related to infarct size (R = 0.12, p = 0.02) and MSI (R = -0.01, p = 0.01) by univariate linear regression analysis with logarithmic transformations, but age had no clinical significance (Fig 4).

**Table 2:** Cardiac Magnetic Resonance Findings according to D-dimer level.

	Overall population	Low D-dimer group ^a^	High D-dimer group ^a^	p value
	n = 208	n = 133	n = 75	
Days of CMR	3.0 (3.0–4.0)	3.0 (3.0–4.0)	4.0 (3.0–5.0)	0.08
Infarct size (% of LV)	20.7 (12.3–28.0)	18.8 (10.7–26.7)	22.3 (16.2–30.5)	0.02
AAR (% of LV)	36.4 (26.4–46.2)	35.8 (24.2–45.3)	38.1 (31.7–46.9)	0.04
MSI	43.8 (31.3–55.9)	47.1 (33.2–57.0)	37.7 (28.2–46.9)	0.01
MVO area (% of LV)	2.3 (0.0–6.8)	2.1 (0.0–6.6)	2.4 (0.0–7.1)	0.65
LVEDV (mL)	143.8 (122.4–165.5)	146.5 (125.4–165.9)	140.0 (119.0–163.1)	0.62
LVESV (mL)	68.0 (50.1–84.4)	68.0 (50.1–83.8)	65.9 (50.1–89.6)	0.31
LVEF (%)	51.9 (45.7–60.1)	53.3 (46.7–60.6)	49.8 (43.9–57.8)	0.02
LV mass (g)	108.1 (91.8–123.6)	112.3 (96.1–128.0)	100.9 (89.3–116.4)	0.01

Values are presented as median (interquartile range) or n (%).

^**a**^Low D-dimer group was defined as D-dimer ≤0.5 μg/mL and high D-dimer group as D-dimer >0.5 μg/mL.

CMR = cardiac magnetic resonance; LV = left ventricle; AAR = area at risk; MSI = myocardial salvage index; MVO = microvascular obstruction; LVEDV = left ventricular end diastolic volume; LVESV = left ventricular end systolic volume; LVEF = left ventricular ejection fraction.

### Predictors of Advanced Myocardial Injury

In multivariable logistic regression analysis, the independent predictors of a large myocardial infarct were high D-dimer levels (odd ratio [OR] comparing high versus low levels 2.59, 95% confidence interval [CI] 1.37 to 4.87; p <0.01), anterior infarct (OR 2.34, 95% CI 1.29 to 4.26; p <0.01), and male sex (OR 2.34, 95% CI 1.14 to 4.80; p = 0.02). High D-dimer level was the only significant independent predictor of lower MSI (OR 2.62, 95% CI 1.44 to 4.78; p <0.01). Manual thrombectomy tended to be related to larger infarct size (OR 1.84, 95% CI 0.99 to 3.44; p = 0.06) and lower MSI (OR 1.68, 95% CI 0.91 to 3.09; p = 0.10), but the differences were not statistically significant (Table 3).

**Table 3:** Multivariable logistic regression analyses for Predictors of Advanced Myocardial Injury.

	Odds Ratio	95% CI	p value
Predictor of Larger Myocardial Infarct (percent infarct volume ≥20%)			
High D-dimer ^a^	2.59	1.37–4.87	<0.01
Anterior infarct	2.34	1.29–4.26	<0.01
Male	2.34	1.14–4.80	0.02
Manual thrombectomy	1.84	0.99–3.44	0.06
Predictor of Lower MSI (MSI <44)			
High D-dimer ^a^	2.62	1.44–4.78	<0.01
Manual thrombectomy	1.68	0.91–3.09	0.10
Dyslipidemia	1.67	0.77–3.59	0.19
Anterior infarct	1.57	0.88–2.81	0.13

^**a**^High D-dimer was defined as D-dimer >0.5 μg/mL.

MSI = Myocardial salvage index; CI = Confidence interval.

## Discussion

We investigated the association between initial blood level of D-dimer on admission and myocardial injury assessed by CMR in patients treated with primary PCI for STEMI. The main finding of our study was that elevated levels of D-dimer on admission were associated with a larger myocardial infarct size and correlated with other CMR parameters, including greater extent of myocardial edema and less myocardial salvage. In multivariate analysis, high D-dimer levels were significantly associated with the risk of larger myocardial infarct and with lower MSI.

The present study supports evidence from previous studies that found an association between elevated D-dimer levels and adverse clinical outcomes. Akgul et al. reported that an elevated blood level of D-dimer on admission in patients undergoing primary PCI for STEMI was a powerful independent predictor of 6-month all-cause mortality [7]. In the Harmonizing Outcomes with Revascularization and Stents in Acute Myocardial Infarction (HORIZONS-AMI) trial, an elevated D-dimer level on admission was associated with adverse cardiovascular events and major bleeding in patients undergoing primary PCI for STEMI [6]. In the HORIZON-AMI trial, D-dimer values were assessed on admission and at discharge, but only D-dimer levels on admission were associated with higher risk of adverse outcomes [6].

CMR provide accurate assessment of myocardial injury including myocardial edema, irreversible MI, and myocardial salvage in reperfused acute MI [18]. In the present study, high D-dimer level on admission predicted larger myocardial infarct and lower MSI, which are correlated with adverse clinical outcomes [9]. Therefore, the results of our study provide a pathological link to support the association of D-dimer levels with adverse outcomes. Elevated D-dimer levels on admission were also associated with a larger AAR assessed by T2-weighted image, likely because a greater extent of thrombus formation and clot burden results in elevated D-dimer levels. In addition, the high D-dimer group had less myocardial salvage than the low D-dimer group. These findings further support the hypothesis that high thrombus burden increases the risk of distal embolization during primary PCI, which in turn may promote infarct expansion. In the present study, the frequency of final myocardial blush grade 3 tended to be lower in the high D-dimer group than in the low D-dimer group. Successful microcirculatory reperfusion, defined angiographically by myocardial blush grade 2 or 3, has been associated with reduction of infarct size and MVO and with better clinical outcomes [19, 20]. These data may also provide a reasonable link between D-dimer level and clinical outcomes.

Our multivariate analyses also showed that anterior infarct and male sex predicted a larger myocardial infarct. It is reasonable that anterior infarcts, which generally affect the largest territory, predict more extensive myocardial injury. A previous meta-analysis using technetium-99m sestamibi imaging [21] and a single-center study using CMR [22] both showed that anterior infarct location was an important predictor of large infarct size. Stone et al. [21] reported that male sex was a predictor of large infarct, and the results of the present study are in accord with the previous report, although the mechanism responsible for this is not obvious. In the present study, further usefulness of D-dimer level was investigated for obtaining additional information on myocardial injury. There might be many confounding factors in this study such as age, gender, current smoker, infarct location, number of diseased vessels, and baseline TIMI flow grade. Especially, age has been known to affect sensitivity and specificity in patients with suspected pulmonary embolism [23]. We analyzed the relationship between age and myocardial injury, however the correlation coefficient for age was low and did not have clinical significance (R = 0.10, p = 0.89 for infarct size, R = -0.05, p = 0.55 for MSI). Age was also eliminated in multivariate logistic regression analysis with a stepwise backward selection process to determine the independent predictors of large myocardial infarct and low MSI.

### Study limitations

This study has several limitations. First, this study was observational study and may be affected by confounding and selection biases. Second, we could only include patients with available CMR, and as a consequence the sample size was not large, which may limit our ability to identify associations. In addition, the generalizability of our findings to may be limited to patients with relatively stable vital signs and modest extent of myocardial injury. Third, age, hepatic or renal function, trauma, infection, stroke, and pulmonary disease, all of which could influence D-dimer level, were not considered in this study. Finally, patients with subclinical deep vein thrombosis may have been included as we did not routinely perform duplex ultrasonography or computed tomographic venography.

## Conclusions

In STEMI patients undergoing primary PCI, high D-dimer levels on admission were associated with a larger myocardial infarct size, a greater extent of AAR, and lower MSI, as assessed by CMR data. Based on our results, elevated initial D-dimer level may be a marker of advanced myocardial injury in patients treated with primary PCI for STEMI. These findings will need to be confirmed in additional large-scale investigations.

## Supporting Information

S1 File Supplementary Appendix. Supplementary Methods.
(DOC)

## References

[1] Dunn KL, Wolf JP, Dorfman DM, Fitzpatrick P, Baker JL, Goldhaber SZ. Normal D-dimer levels in emergency department patients suspected of acute pulmonary embolism. Journal of the American College of Cardiology. 2002;40(8):1475–8. Epub 2002/10/24. . 12392839 10.1016/s0735-1097(02)02172-1

[2] Virmani R, Burke AP, Farb A, Kolodgie FD. Pathology of the vulnerable plaque. Journal of the American College of Cardiology. 2006;47(8 Suppl):C13–8. Epub 2006/04/25. 10.1016/j.jacc.2005.10.065 . 16631505

[3] Oldgren J, Linder R, Grip L, Siegbahn A, Wallentin L. Coagulation activity and clinical outcome in unstable coronary artery disease. Arteriosclerosis, thrombosis, and vascular biology. 2001;21(6):1059–64. Epub 2001/06/09. . 11397720 10.1161/01.atv.21.6.1059

[4] Ridker PM, Hennekens CH, Cerskus A, Stampfer MJ. Plasma concentration of cross-linked fibrin degradation product (D-dimer) and the risk of future myocardial infarction among apparently healthy men. Circulation. 1994;90(5):2236–40. Epub 1994/11/01. . 7955179 10.1161/01.cir.90.5.2236

[5] Menown IB, Mathew TP, Gracey HM, Nesbitt GS, Murray P, Young IS, et al Prediction of Recurrent Events by D-Dimer and Inflammatory Markers in Patients with Normal Cardiac Troponin I (PREDICT) Study. American heart journal. 2003;145(6):986–92. Epub 2003/06/11. 10.1016/s0002-8703(03)00169-8 . 12796753

[6] Kikkert WJ, Claessen BE, Stone GW, Mehran R, Witzenbichler B, Brodie BR, et al D-dimer levels predict ischemic and hemorrhagic outcomes after acute myocardial infarction: a HORIZONS-AMI biomarker substudy. Journal of thrombosis and thrombolysis. 2014;37(2):155–64. Epub 2013/08/09. 10.1007/s11239-013-0953-5 . 23925451

[7] Akgul O, Uyarel H, Pusuroglu H, Gul M, Isiksacan N, Turen S, et al Predictive value of elevated D-dimer in patients undergoing primary angioplasty for ST elevation myocardial infarction. Blood coagulation & fibrinolysis: an international journal in haemostasis and thrombosis. 2013;24(7):704–10. Epub 2013/04/11. 10.1097/MBC.0b013e3283610396 . 23571687

[8] Dall'Armellina E, Karamitsos TD, Neubauer S, Choudhury RP. CMR for characterization of the myocardium in acute coronary syndromes. Nature reviews Cardiology. 2010;7(11):624–36. Epub 2010/09/22. 10.1038/nrcardio.2010.140 . 20856263

[9] Eitel I, de Waha S, Wohrle J, Fuernau G, Lurz P, Pauschinger M, et al Comprehensive prognosis assessment by CMR imaging after ST-segment elevation myocardial infarction. Journal of the American College of Cardiology. 2014;64(12):1217–26. Epub 2014/09/23. 10.1016/j.jacc.2014.06.1194 . 25236513

[10] Froehling DA, Daniels PR, Swensen SJ, Heit JA, Mandrekar JN, Ryu JH, et al Evaluation of a quantitative D-dimer latex immunoassay for acute pulmonary embolism diagnosed by computed tomographic angiography. Mayo Clinic proceedings. 2007;82(5):556–60. Epub 2007/05/12. 10.4065/82.5.556 . 17493420

[11] Song YB, Hahn JY, Gwon HC, Chang SA, Lee SC, Choe YH, et al A high loading dose of clopidogrel reduces myocardial infarct size in patients undergoing primary percutaneous coronary intervention: a magnetic resonance imaging study. American heart journal. 2012;163(3):500–7. Epub 2012/03/20. 10.1016/j.ahj.2011.12.007 . 22424023

[12] Lonborg J, Vejlstrup N, Kelbaek H, Holmvang L, Jorgensen E, Helqvist S, et al Final infarct size measured by cardiovascular magnetic resonance in patients with ST elevation myocardial infarction predicts long-term clinical outcome: an observational study. European heart journal cardiovascular Imaging. 2013;14(4):387–95. Epub 2012/11/28. 10.1093/ehjci/jes271 . 23178864

[13] Eitel I, Desch S, Fuernau G, Hildebrand L, Gutberlet M, Schuler G, et al Prognostic significance and determinants of myocardial salvage assessed by cardiovascular magnetic resonance in acute reperfused myocardial infarction. Journal of the American College of Cardiology. 2010;55(22):2470–9. Epub 2010/06/01. 10.1016/j.jacc.2010.01.049 . 20510214

[14] Rezkalla SH, Kloner RA. No-reflow phenomenon. Circulation. 2002;105(5):656–62. Epub 2002/02/06. . 11827935 10.1161/hc0502.102867

[15] Croisille P, Kim HW, Kim RJ. Controversies in cardiovascular MR imaging: T2-weighted imaging should not be used to delineate the area at risk in ischemic myocardial injury. Radiology. 2012;265(1):12–22. Epub 2012/09/21. 10.1148/radiol.12111769 . 22993217

[16] Chung S, Song YB, Hahn JY, Chang SA, Lee SC, Choe YH, et al Impact of white blood cell count on myocardial salvage, infarct size, and clinical outcomes in patients undergoing primary percutaneous coronary intervention for ST-segment elevation myocardial infarction: a magnetic resonance imaging study. The international journal of cardiovascular imaging. 2014;30(1):129–36. Epub 2013/10/10. 10.1007/s10554-013-0303-x . 24104952

[17] Ganame J, Messalli G, Dymarkowski S, Rademakers FE, Desmet W, Van de Werf F, et al Impact of myocardial haemorrhage on left ventricular function and remodelling in patients with reperfused acute myocardial infarction. European heart journal. 2009;30(12):1440–9. Epub 2009/04/07. 10.1093/eurheartj/ehp093 . 19346229

[18] Friedrich MG, Abdel-Aty H, Taylor A, Schulz-Menger J, Messroghli D, Dietz R. The salvaged area at risk in reperfused acute myocardial infarction as visualized by cardiovascular magnetic resonance. Journal of the American College of Cardiology. 2008;51(16):1581–7. Epub 2008/04/19. 10.1016/j.jacc.2008.01.019 . 18420102

[19] Henriques JP, Zijlstra F, van 't Hof AW, de Boer MJ, Dambrink JH, Gosselink M, et al Angiographic assessment of reperfusion in acute myocardial infarction by myocardial blush grade. Circulation. 2003;107(16):2115–9. Epub 2003/04/16. 10.1161/01.cir.0000065221.06430.ed . 12695301

[20] Brener SJ, Maehara A, Dizon JM, Fahy M, Witzenbichler B, Parise H, et al Relationship between myocardial reperfusion, infarct size, and mortality: the INFUSE-AMI (Intracoronary Abciximab and Aspiration Thrombectomy in Patients With Large Anterior Myocardial Infarction) trial. JACC Cardiovascular interventions. 2013;6(7):718–24. Epub 2013/07/20. 10.1016/j.jcin.2013.03.013 . 23866184

[21] Stone GW, Dixon SR, Grines CL, Cox DA, Webb JG, Brodie BR, et al Predictors of infarct size after primary coronary angioplasty in acute myocardial infarction from pooled analysis from four contemporary trials. The American journal of cardiology. 2007;100(9):1370–5. Epub 2007/10/24. 10.1016/j.amjcard.2007.06.027 17950792

[22] Hahn JY, Song YB, Gwon HC, Choe YH, Kim JH, Sung J, et al Relation of left ventricular infarct transmurality and infarct size after primary percutaneous coronary angioplasty to time from symptom onset to balloon inflation. The American journal of cardiology. 2008;102(9):1163–9. Epub 2008/10/23. 10.1016/j.amjcard.2008.06.042 . 18940285

[23] Righini M, Goehring C, Bounameaux H, Perrier A. Effects of age on the performance of common diagnostic tests for pulmonary embolism. The American journal of medicine. 2000;109(5):357–61. Epub 2000/10/06. . 11020391 10.1016/s0002-9343(00)00493-9

