# Supplementary material for: D-Dimer Levels Predict Myocardial Injury in ST-Segment Elevation Myocardial Infarction: A Cardiac Magnetic Resonance Imaging Study
Source: PLoS One. 2016 Aug 11;11(8):e0160955. doi: 10.1371/journal.pone.0160955 (PMC4981325; doi:10.1371/journal.pone.0160955)
Supplement: S1 File — Supplementary Methods. (DOC) [file pone.0160955.s001.doc]

**S1. Supplementary Appendix**

**(1) Supplementary Methods**

**Percutaneous Coronary Intervention**

All coronary interventions were performed according to current standard procedural guidelines. All patients received a loading dose of aspirin (300 mg) and clopidogrel (300 or 600 mg) before primary percutaneous coronary intervention (PCI), unless they had previously received these antiplatelet medications. The anticoagulants were administered during PCI based on the current practice guidelines established by the Korean Society of Interventional Cardiology. Drug-eluting stents were used without restriction, and the duration of dual antiplatelet therapy was determined by the operators. Myocardial blush grade (MBG) was evaluated using the final angiogram, defined previously as follows: 0, no myocardial blush or contrast density; 1, minimal myocardial blush or contrast density; 2, moderate myocardial blush or contrast density less than that obtained during angiography of a contralateral or ipsilateral non-infarct-related coronary artery; and 3, normal myocardial blush or contrast density, comparable with that obtained during angiography of a contralateral or ipsilateral non-infarct-related coronary artery . All baseline and procedural cine coronary angiograms were reviewed and analyzed at the institution’s angiographic core laboratory.

**Cardiac Magnetic Resonance Protocol**

Cardiac magnetic resonance (CMR) imaging was performed using a 1.5-T magnetic scanner (Achieva, Philips Medical Systems, Best, Netherlands) with a SENSE cardiac coil based on our laboratory protocol . The CMR protocol included cine, T2-weighted, first-pass perfusion, and late-gadolinium enhancement imaging. CMR images were obtained using a fast gradient echo sequence (balance fast field echo, steady-state free precession) along the long and the short axes, which extended from the apex to the base of the left ventricle. T2-weighted magnetic resonance imaging was performed in the cardiac short-axis direction using a dark-blood T-2 weighted inversion-recovery fast-spin echo sequence. Next, 0.15 mmol/kg gadolinium-diethylenetriamine pentaacetic acid (DTPA-Gd, Magnevist; Bayer Schering Pharma, Berlin, Germany) was injected intravenously at a rate of 3 mL/s, followed by a saline flush. The T1-weighted dynamic sequence enabled a first pass of the contrast agent through the myocardium after intravenous bolus injection (turbo field echo with SENSE, repetition time/echo time, 2.6/1.3 milliseconds). The slice thickness was 6 mm, with a field of view of 40 x 40 cm and an image matrix of 128 x 128 for the first-pass perfusion study. Images were obtained for 40 phases at each of the four locations for every two heart beats. Delayed hyperenhancement and extent of microvascular obstruction (MVO) were evaluated at 5, 10, and 15 minutes after DTPA-Gd infusion in 10 to 12 contiguous, 6 mm slices. The measurements were made with a 4-mm interslice gap using a multishot turbo field echo breath-hold sequence, with a nonselective inversion (typical repetition time/time to echo 4.6/1.4 milliseconds). The field of view and image matrix were 35 x 35 cm and 256 x 256, respectively. The inversion delay time ranged between 200 and 300 milliseconds. A Look-Locker sequence was used to determine the optimal inversion time.

**References**

1. van 't Hof AW, Liem A, Suryapranata H, Hoorntje JC, de Boer MJ, Zijlstra F. Angiographic assessment of myocardial reperfusion in patients treated with primary angioplasty for acute myocardial infarction: myocardial blush grade. Zwolle Myocardial Infarction Study Group. Circulation. 1998;97(23):2302-6. Epub 1998/06/25. PubMed PMID: 9639373.

2. Song YB, Hahn JY, Gwon HC, Chang SA, Lee SC, Choe YH, et al. A high loading dose of clopidogrel reduces myocardial infarct size in patients undergoing primary percutaneous coronary intervention: a magnetic resonance imaging study. American heart journal. 2012;163(3):500-7. Epub 2012/03/20. doi: 10.1016/j.ahj.2011.12.007. PubMed PMID: 22424023.
